# Integration of contraception provision in emergency obstetric and neonatal care: A scoping review

**DOI:** 10.1002/ijgo.70768

**Published:** 2026-01-05

**Authors:** Mikaela R. Koch, Asha Kasliwal, Nasser Elkholy, Kidist Gizachew, Azra Ashan, Anita Makins, Helena Kallner, Sian Mosedale, Aparna Sridhar

**Affiliations:** ^1^ David Geffen School of Medicine Los Angeles California USA; ^2^ Manchester University NHS Foundation Trust Manchester UK; ^3^ Department of Obstetrics and Gynecology Ain Shams University Cairo Egypt; ^4^ St. Paul's Hospital Millennium Medical College Addis Ababa Ethiopia; ^5^ Association for Mothers and Newborns Karachi Pakistan; ^6^ Nuffield Department of Women's and Reproductive Health University of Oxford Oxford UK; ^7^ Department of Obstetrics and Gynecology Danderyds Hospital Stockholm Sweden

**Keywords:** contraception, emergency obstetric and neonatal care, emergency obstetrics, family planning, integration, scoping review

## Abstract

**Background:**

Emergency obstetric and neonatal care (EmONC) provides a framework to assess the capacity of health systems and mitigate maternal mortality. Given the high unmet need for contraception, integrating contraception provision and EmONC services could improve maternal and newborn health outcomes.

**Objectives:**

This scoping review aimed to examine the integration of contraceptive and EmONC services and identify opportunities for future work in this space.

**Method:**

A comprehensive search strategy was developed with sexual reproductive health and rights (SRHR) experts and librarians incorporating EmONC and contraceptive‐specific terms. PubMed, EMBASE, CINAHL, Web of Science, and Cochrane Library were searched. Studies published before 2000 or those not in English were excluded. Nine reviewers conducted screening and full text reviews using Covidence with conflicts resolved through biweekly meetings. Data was analyzed descriptively.

**Results:**

Of 7105 screened articles, 28 ultimately met inclusion criteria. Most were retrospective or cross‐sectional in design and conducted in hospital settings. Fourteen studies explicitly referenced integration of contraception and EmONC services primarily at the time of emergency cesarean or incomplete abortions. Copper intrauterine devices and permanent sterilization were the most common methods provided. Barriers to integration included cultural stigma, routine deferral to outpatient settings, provider hesitation and gaps in training. Facilitators proposed included staff training, antenatal contraceptive counseling, and improved interprofessional communication.

**Conclusion:**

Given the dearth of literature on contraception in conjunction with EmONC services, there is a demonstrated need to standardize integration within EmONC indicators, metrics, and classifications. Strengthening provider training and institutional policies at national and international levels could enhance integration and improve outcomes.

## 1 INTRODUCTION

Emergency obstetric and neonatal care (EmONC) was first introduced by the World Health Organization (WHO), the United Nations Children's Fund (UNICEF), and the United Nations Population Fund (UNFPA) in 1997.^1^ As a set of indicators describing necessary resources or abilities to treat and prevent obstetric and neonatal emergencies, EmONC provides a framework for governments and the international community to assess, and understand, the capacity of the existing health systems to respond to the reproductive health needs of their population.^2^, ^3^ Thus, EmONC is a set of criteria and standards for healthcare provision in emergency obstetric and neonatal contexts. It is a critical metric allowing governments and international stakeholders to assess and build healthcare systems globally that can be held accountable.

EmONC is divided into two categories: basic EmONC (BEmONC) consisting of interventions that can be provided in primary healthcare facilities or community settings. It includes seven “signal functions”: neonatal resuscitation, parenteral antibiotics, magnesium sulphate for the treatment of eclampsia, manual vacuum aspiration of retained products, manual placental removal, uterotonic administration and assisted vaginal delivery. Comprehensive EmONC (CEmONC) can be provided at larger hospitals or referral facilities and includes two additional signal functions: the capacity to provide both cesarean sections and blood transfusions (Table 1).^2^ According to the WHO, countries have appropriate infrastructure to manage pregnancy‐related complications if there are five facilities per 500 000 inhabitants that have provided all BEmONC signal functions within the past 3 months.^4^ EmONC training and standards have shown to be effective in improving maternal and neonatal outcomes, with one study in Kenya finding that postpartum hemorrhage rates decreased from 3.5% to 2.3% after an EmONC program was implemented.^5^ Another study in Tanzania found that postpartum hemorrhage rates decreased from 32.9% to 18.2% after initiation of EmONC training.^6^ Moreover, some have argued that reduced availability or low‐quality EmONC services is the single most important factor implicated in maternal deaths in conflict and post‐conflict zones.^7^

**TABLE 1:** Emergency obstetric and neonatal care indicators.^2^

Basic services	Comprehensive services
(1) Administer parenteral antibiotics	Perform signal functions 1–7 plus
(2) Administer uterotonic drugs (i.e., parenteral oxytocin)	(8) Perform surgery (e.g., cesarean section)
(3) Administer parenteral anticonvulsants for pre‐eclampsia and eclampsia (i.e., magnesium sulfate)	(9) Perform blood transfusion
(4) Manually remove the placenta	
(5) Remove retained products (e.g., manual vacuum extraction, dilation, and curettage)	
(6) Perform assisted vaginal delivery (e.g., vacuum extraction and forceps delivery)	
(7) Perform basic neonatal resuscitation (e.g., with bag and mask)	

*Note:* A basic emergency obstetric care facility is one in which all functions 1–7 are performed. A comprehensive emergency obstetric care facility is one in which all functions 1–9 are performed.

Although it is clear that contraception and EmONC services are one continuum, provision of contraception is not a direct component of the core EmONC classification, indicators, or metrics. Enhancing the availability of contraceptive services improves maternal, neonatal, and childhood health, prevents unintended pregnancies, and lengthens inter‐pregnancy intervals which, in turn, reduce the demand for EmONC.^8^, ^9^ Short interpregnancy intervals following an obstetric emergency can lead to high maternal morbidity and using contraception to help facilitate safer interpregnancy intervals not only helps to improve outcomes but provides opportunities for further counseling.^10^, ^11^ It is estimated that contraception alone could reduce maternal mortality by 30%.^12^ It is also clear that providing EmONC and contraception services together exponentially improves outcomes. One study found that improving access to midwifery‐led comprehensive family planning interventions, including contraception in addition to EmONC interventions, could avert up to 83% of maternal and neonatal deaths.^13^ Moreover, many EmONC requirements have direct implications for contraception, such as improving supply chains to address EmONC indicators concurrently improving accessibility of contraceptive options.^14^

In some countries, such as Afghanistan, South Sudan, and Ethiopia, contraception is increasingly being incorporated into EmONC training.^15^, ^16^, ^17^ The most direct integration has been seen with post‐abortion contraception. The Ethiopian Ministry of Health, for example, has undertaken initiatives through their Health Extension Program to ensure that health centers offer both BEmONC signal functions, contraceptive services, and post‐abortion care.^18^ Bangladesh and Guinea have also both created policies to ensure post abortion care was made accessible at all BEmONC facilities, including appropriate linkage and referral mechanisms if advanced technology or management was needed.^19^ And yet, for the most part, contraceptive counseling and delivery remain absent from core EmONC indicators, metrics, and classifications, and even more broadly discussions around EmONC.

Given the exploratory nature of our question and the variability we anticipated regarding the types of studies and volume of studies available, a scoping review methodology was chosen. This allowed us to assess both the breadth and depth of available evidence without being constrained to a narrowly defined research question.

The objective of this scoping review was to search the literature for data on the direct integration of contraception and EmONC services with the intention of identifying gaps in the implementation of reproductive health services and provide opportunities and recommendations for future work in this space.

## 2 METHODS

Our protocol was drafted using Joanna Briggs Institute methodology and was reviewed and revised by research leads and consulting University of California Los Angeles librarians.^20^ The protocol is not publicly available. This study was a scoping review. Unlike a systematic review, this study was designed to map concepts and research gaps in the field rather than to synthesize outcomes or provide definitive answers or statistics focused on a specific clinical question. Accordingly, no formal quality appraisal or statistical analysis was performed, consistent with scoping review guidelines.

### 2.1 Information sources

Databases included in the study were PubMed, EMBASE, CINAHL, Web of Science, and Cochrane Library. These databases were selected to include a comprehensive set of health sciences and medical sources, including both those from medical and nursing domains.

### 2.2 Search Strategy

An initial limited search in PubMed and Google Scholar was undertaken to identify articles on the topic. The text words contained in the titles and abstracts of relevant articles, and the index terms used to describe the articles were used to develop the full search strategy (Appendix A). The search strategy included words in the contraceptive space, including “contraceptive pill” and “intrauterine device” and in the EmONC sphere, including “neonatal resuscitation,” and “hemorrhage.” The search strategies were developed in consultation with an experienced librarian and refined through team discussions.

### 2.3 Data extraction

Databases were searched on April 8, 2024, using the study search strategy (Appendix A). The final search results were exported into Covidence, and duplicates were removed.

### 2.4 Study selection

A team of nine reviewers subsequently completed an initial abstract screening. Every article was evaluated by two independent reviewers and the team met biweekly to discuss questions and resolve conflicts.

**FIGURE 1:**
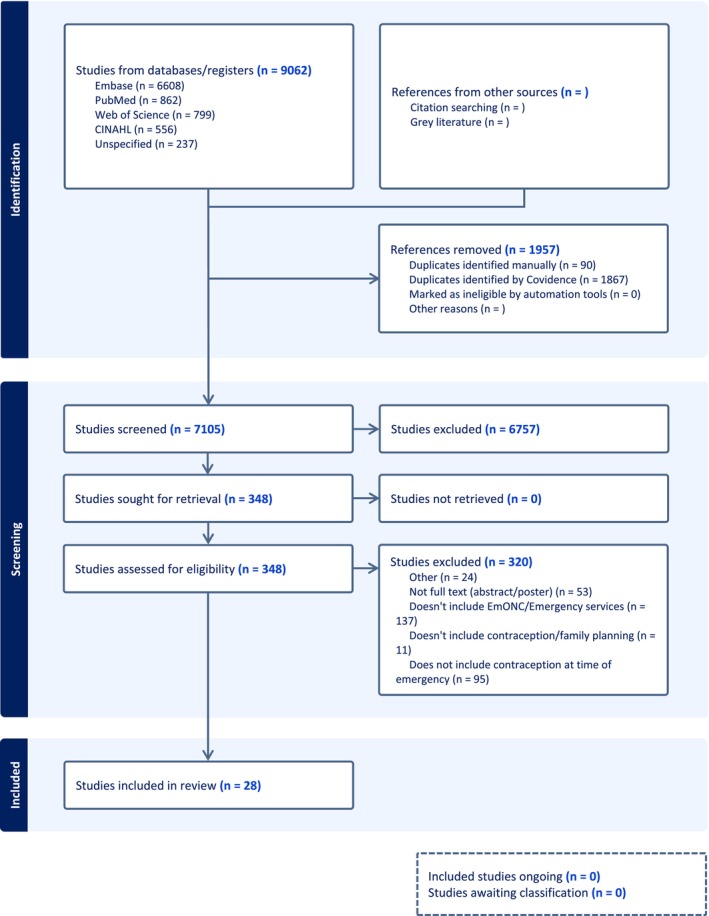
PRISMA flow diagram for scoping review sources.

Full texts were then imported into Covidence and screened by a set of five reviewers. All articles were screened by two independent reviewers with a third reviewer resolving conflicts. Reasons for exclusion were documented (Figure 1). Any disagreements that arose between reviewers were resolved through discussion with the rest of the reviewing team.

### 2.5 Eligibility criteria

To be included in the study, articles needed to measure or focus on specific opportunities or examples of contraceptive integration at the time of obstetric or neonatal emergency or indicate co‐implementation of contraception and EmONC initiatives. Studies that were published after 2000 and were in English were included. The year 2000 was chosen as a cutoff year to reflect when the term “EmONC,” initially coined in 1997, was likely to have started appearing in the international global health rhetoric.

In summary, all publication types, including primary research studies, letters, guidelines, systematic reviews, and qualitative and quantitative documents, were included in this review. There were no limitations on patient population or geographic location of included studies, outside the requirement that they be in English and published between 2000 and 2024. Case reports or case series were excluded from the study.

### 2.6 Data analysis

A data extraction tool was developed jointly by two reviewers to determine which variables to extract, focused specifically on information regarding integration (Appendix B). Data was extracted by two reviewers, and the data extraction tool was updated iteratively as necessary.

We extracted data on country of origin, setting, sample size, population characteristics, nature of obstetric emergency, scenario and type of contraceptive, and facilitators and barriers to this integration.

### 2.7 Data synthesis

The quantitative data was analyzed and reported descriptively with overall percentages, grouping studies by type of emergency and nature of integration. Qualitative data was analyzed and presented thematically. Conclusions and implications for research and practice were highlighted.

## 3 RESULTS

**FIGURE 2:**
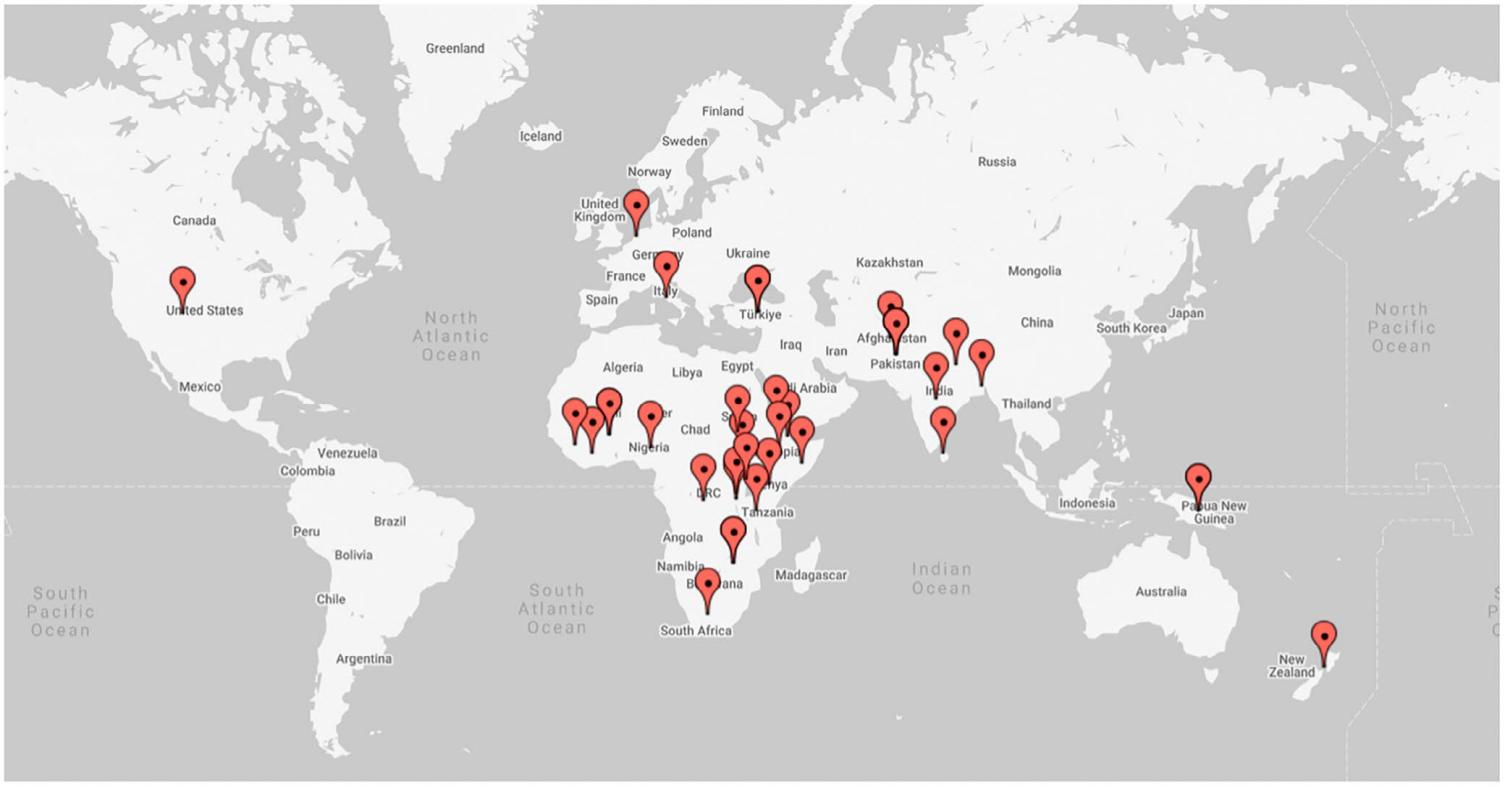
Map of included studies' geographical origin.

Of 7105 screened articles, 28 ultimately met the inclusion criteria (Figure 1 and Table 2). Included articles were published between 2003 and 2024, with studies and reports from 30 countries (Figure 2).

**TABLE 2:** Literature sources identified in scoping review (*n* = 28).

Author/Title	Year	Location	Study design	EmONC service	Contraceptive service	Study objective
Remez ^21^ “Offering a woman sterilization during an emergency cesarean section may sometimes be appropriate”	2003	Zimbabwe	Observational, retrospective study	Emergency C‐section	Tubal ligation	Those undergoing emergency C‐section were offered and provided tubal ligation at time of C‐section
Compaore ^22^ “Availability and Utilization of Postabortion Care Services in Burkina Faso, Côte d'Ivoire, and Guinea: A Secondary Analysis of Emergency Obstetric and Neonatal Care Needs Assessments (EmONC).”	2022	Burkina Faso, Ivory Coast, & Guinea	Cross‐sectional	Abortion services	General contraceptive provision	Offering and providing contraceptive services to women following treatment for abortion‐related complications
Esegwui ^23^ “Sterilization at cesarean section in Nigeria”	2004	Nigeria	Observational, retrospective study	Emergency C‐section	Tubal ligation	Study on all patients who underwent tubal ligation at the time of cesarean delivery, including those who underwent emergency C‐section
Healy ^24^ “Counting abortions so that abortion counts: Indicators for monitoring the availability and use of abortion care services”	2006	Not site based (conceptual paper)	Conceptual paper	Abortion services	General contraceptive provision	Study proposing a set of indicators to a comprehensive approach to measuring whether abortion‐related needs are met, including a recommendation to train and utilize healthcare professionals to counsel and provide contraceptives following uterine evacuation for abortion and abortion‐related complications
Mapira ^25^ “The contribution of church health services to maternal health care provision in Papua New Guinea.”	2011	Papua New Guinea	Descriptive analysis	Health facility capacity for EmONC	General contraceptives	Aim to provide a national snapshot of the provision of maternal and reproductive health care services through the church, providing an implementation plan for integrating contraceptives into the health system with emergency services
Bhutta ^26^ “Insertion of intrauterine contraceptive device at cesarean section”	2011	Pakistan	Case–control study	Emergency C‐section	Copper IUD	Goal to determine safety of IUCD at time of CS, including cases of emergency C section
Gilmore ^27^ “What will it take to eliminate preventable maternal deaths?”	2012	Not site based (conceptual paper)	Conceptual paper	EmONC in policy context	General contraceptives	Opinion text discussing interventions and responses needed to eliminate maternal mortality, including ensuring family planning and BeMONC be provided at every community facility and health center
Mazhar ^28^ “Obstetric services in Pakistan: Where are we and what is the way forward?”	2013	Pakistan	Conceptual paper	EmONC in policy context	General contraceptives	Narrative piece indicating the importance of having emergency and comprehensive care for delivery and preventative services like FP available under the same roof
Arambepola ^29^ “Usual hospital care versus post‐abortion care for women with unsafe abortion: a case control study from Sri Lanka”	2014	Sri Lanka	Case–control study	Abortion services	Contraceptive counseling	Study comparing post‐abortion contraception given to women following unsafe abortion versus routine hospital care following spontaneous abortion
Sucak ^30^ “Immediate postplacental insertion of a copper intrauterine device: A pilot study to evaluate expulsion rate by mode of delivery”	2015	Turkey	Cohort study	Emergency C‐section	Copper IUD	Study comparing IUD expulsion rate in vaginal versus CD, including those undergoing emergency C‐section.
Huda ^31^ “Strengthening health systems capacity to monitor and evaluate programmes targeted at reducing abortion‐related maternal mortality in Jessore district, Bangladesh”	2015	Bangladesh	Non‐randomized experimental study	Post abortion	General contraceptives	Project aimed at implementing a set of indicators based on the safe abortion care model and indicators for monitoring emergency obstetric interventions
MacDonald ^32^ “Contraception post severe maternal morbidity: A retrospective audit”	2015	New Zealand	Retrospective study	Women with severe acute maternal morbidity	Contraceptive counseling	This study examined the contraceptive advice and prescription for severe acute maternal morbidity cases with or without severe preexisting comorbidity
Arlier ^33^ “Tubal sterilization during cesarean section at a training hospital in Turkey: A clinical and demographic analysis”	2018	Turkey	Cross sectional study	Emergency C‐section	Tubal ligation	Study assessing short‐term postoperative effects in patients undergoing BTL during C‐section versus those who did not
Ferrari ^34^ “Opportunistic salpingectomy during postpartum contraception procedures at elective and unscheduled cesarean delivery”	2019	Italy	Retrospective audit	Emergency C‐section	Tubal ligation	Study comparing intra‐ and postoperative surgical complications of opportunistic bilateral total salpingectomy during postpartum permanent contraception procedures in elective and unscheduled cesarean delivery
Escobar ^35^ “Immediate Postpartum Contraception: Intrauterine Device Insertion”	2019	International	Conceptual paper	Emergency C section	IUD – copper/hormonal	Article providing an overview of immediate postpartum IUD insertion, risks and benefits, and eligibility criteria and describes pre‐insertion, insertion, and postinsertion care
Jakhar ^36^ “Safety and Efficacy of Intra‐cesarean IUCD: A Prospective Study at a Tertiary Care Centre”	2019	India	Cohort study	Emergency C‐section	Copper IUD	Evaluate the efficacy and complications of intra‐cesarean PPIUD insertion
Glover ^37^ “Assessing Readiness to Provide Comprehensive Abortion Care in the Democratic Republic of the Congo After Passage of the Maputo Protocol”	2020	Democratic Republic of the Congo	Cross‐sectional study	Post abortion	Copper IUD and hormonal contraceptives	Assessing readiness to provide abortion care as including contraceptive provision following unsafe abortion
Schutte ^38^ “A review of intrauterine device placement during cesarean section at level two facilities in the Metro West, Cape Town”	2021	South Africa	Retrospective audit	Emergency C‐section	Copper IUD	Assess the practice of placement of IUDs at C‐section and describe follow‐up
Maruf ^39^ “Health facility capacity to provide postabortion care in Afghanistan: a cross‐sectional study”	2021	Afghanistan	Cross‐sectional study	Post abortion	Contraceptive counseling and general contraceptive provision	Assess the capacity of health facilities to provide post abortion care services including contraceptives
Sinkey ^40^ “The effects of offering immediate postpartum placement of IUDs and implants to pregnant patients with heart disease”	2022	United States	Retrospective cohort study	Immediate postpartum contraception	Copper/hormonal IUD	Examine LARC utilization among pregnant patients before and after implementation of the immediate postpartum LARC program
Hou ^41^ “Readiness of health facilities to provide emergency obstetric care in Papua New Guinea: Evidence from a cross‐sectional survey”	2022	Papa New Guinea	Cross‐sectional study	Health facility capacity for EmONC	General contraceptives	To measure health facility readiness to provide obstetric care and other maternal health services, including family planning readiness and emergency readiness
Chaudary ^42^ “Role of Postpartum IUCD Insertion after C‐Section”	2022	Pakistan	Randomized controlled trial	Emergency C‐section	Copper IUD	To assess safety and complications with post‐cesarean IUCD
Rai ^43^ “Health facility availability and readiness for family planning and maternity and neonatal care services in Nepal: Analysis of cross‐sectional survey data”	2023	Nepal	Cross‐sectional study	Health facility capacity for EmONC	General contraceptives	To determine the availability and readiness of health facilities to provide family planning, antenatal care, and basic emergency obstetric and newborn care in Nepal
Onadja ^44^ “Postabortion care service availability, readiness, and access in Burkina Faso: results from linked female‐facility cross‐sectional data”	2024	Burkina Faso	Cross‐sectional study	Post abortion	Contraceptive counseling and general contraceptive provision	To evaluate post‐abortion care services available and assess readiness to provide
Bergevin ^45^ “Towards ending preventable maternal deaths by 2035”	2015	International	Conceptual paper	EmONC in policy context	General contraceptives	To examine scenarios that could reduce maternal mortality by 2035
van Boekholt ^46^ “Review of maternal death audits in refugee camps in UNHCR East and Horn of Africa and Great Lakes Region, 2017–2019”	2023	Burundi, Djibouti, Eritrea, Ethiopia, Kenya, Rwanda, Somalia, South Sudan, Sudan, Tanzania, and Uganda	Retrospective audit study	Postpartum hemorrhage	General contraceptives	To assess the quality of maternal death audits, causes of maternal deaths, and identify barriers in maternal care in refugee settings
Verkuyl ^47^ “Sterilization during unplanned cesarean sections for women likely to have a completed family – Should they be offered? Experience in a country with limited health resources”	2002	Zimbabwe	Cohort study	Emergency C‐section	Tubal ligation	To determine if it is appropriate to give a woman of higher parity who needs a C‐section at short notice the option of a tubal ligation
Verkuyl ^48^ “The right to informed choice. A study and opinion poll of women who were or were not given the option of a sterilization with their cesarean section”	2011	Netherlands	Cross‐sectional study	Emergency C‐section	Tubal ligation	To determine the views and experiences of patients with informed choice about the option of C‐section/TO during pregnancy

Abbreviations: BTL, bilateral tubal ligation; CD, cesarean delivery; IUD, intrauterine devices; LARC, long‐acting reversible contraception; PPIUD, postpartum intrauterine device.

Included studies ranged from randomized control trials to opinion pieces, with most studies retrospective or cross‐sectional in design (Table 2). Of the included articles, the majority were studies conducted in hospital settings, while the remainder included settings such as community health units, aid posts, private healthcare facilities, and refugee camps.

Fourteen of the articles referenced direct integration of contraceptive services with the sample size of participants included ranging from two to 418. These articles were most commonly studies assessing copper intrauterine device (IUCD) insertion or tubal ligation at the time of emergency cesarean section or contraceptive counseling/provision for those with incomplete or unsafe abortions (Table 2). Those that did not reference integration directly were commonly opinion or narrative pieces discussing recommendations or hypothetical plans for integration, often emphasizing the importance of ensuring hospital systems had both EmONC and contraceptive services available in tandem.

### 3.1 Barriers

When reviewing the included articles for discussions around barriers to the integration of contraception and EmONC services obstacles at structural, provider, and patient levels were noted.

### 3.2 Structural

At the facility level, contraceptive services were at times unavailable due to stock outs or logistical challenges.^37^ Financial constraints were identified as a barrier along with commodity shortages inhibiting access and hard‐to‐reach facilities, and some settings noted that EmONC guidelines were either unavailable or inconsistently implemented.^27^, ^39^, ^46^

### 3.3 Provider

Scope of practice and workflow limitations were highlighted barriers in multiple articles as well as poor communication between care facilities.^46^ According to the articles, some providers preferred to defer contraceptive discussions to outpatient settings or midwives felt contraceptive provision was outside their scope of practice.^29^, ^32^, ^44^ However, the consistency of contraceptive counseling and provision in outpatient settings, as well as patient attendance at these follow‐up appointments, was unclear. Articles also noted that there were some barriers at the provider level due to reluctance to offer intrauterine devices (IUD) at time of emergency cesarean section due to perceived time constraints and inability to consult family members.^33^, ^36^, ^48^ Further, provider knowledge and training were highlighted as a limitation with low provider motivation and inadequate training.^39^, ^40^, ^46^

### 3.4 Patient

Additional personal barriers at both provider and patient levels included stigma associated with contraception and religious and cultural beliefs influencing contraceptive decision‐making such as requiring spousal consultation before contraceptive initiation or norms around sterilization procedures.^24^, ^25^, ^40^ Some articles also mentioned low baseline contraceptive uptake even in facilities with adequate capacity, suggesting gaps in demand generation and service delivery.^24^

### 3.5 Facilitators

Despite these barriers, several facilitators were identified as either successful for enhancing integration of contraception and EmONC services or as future recommendations.

Some key strategies proposed included increasing financial accessibility by subsidizing services and addressing counseling during antenatal visits with the aim of reducing provider hesitation around consenting for intraoperative contraception during emergency cesarean section.^36^, ^37^ Additionally, regular management meetings and quality improvement activities were noted to help strengthen service delivery.^44^ Further, specific interventions including improving interpersonal provider communication and expanding the scope of health professionals able to provide MVA's allowed for increased opportunity and access for simultaneous contraceptive provision. ^25^

Community‐based strategies included home visits by fieldworkers, ensuring experienced physicians were providing and counseling on contraceptive uptake and provider training always includes both contraceptive services and essential EmONC interventions.^22^, ^30^

### 3.6 Additional findings

There were several notable article‐specific findings relevant to the discussion regarding EmONC and contraceptive integration.

Specifically, one study found that bilateral tubal ligation (BTL) as an option was not discussed during pregnancy among patients planning to undergo vaginal birth after cesarean in 84% of the cases despite the increased risk of delivering via cesarean section for those women.^49^ In other studies that followed women who had an emergency cesarean section (CS), of those that were offered BTL, 8% regretted declining it, 2% regretted accepting it, and 1% expressed dissatisfaction due to a clinical oversight in performing the procedure, overall finding a high acceptability rate for BTL during emergency CS.^21^, ^49^ That being said, given the irreversible nature of the procedure, these authors believe the importance of including such counseling in the antenatal period whenever possible should be emphasized.

Another study found that when looking at individuals who underwent unsafe abortions, which often requires manual vaccum aspiration (MVA) technique, they were less likely to receive contraceptive counseling than controls and did not receive any formal contraceptive counseling during their inpatient stay.^26^, ^31^ It was noted that the timing of provision of contraception as part of EmONC services does not help prevent the current unplanned pregnancy, only subsequent ones. Hence, it was suggested that EmONC, postpartum care, postabortion care, and preventative services such as contraception should ideally be integrated within the same facility to ensure full coverage and continuity of care.^26^, ^30^

Regarding safety and effectiveness of the integration, multiple studies assessing IUCD or BTL at time of emergency section found it to be safe and effective.^32^, ^35^, ^43^ One study found that those who participated in active labor prior to emergency cesarean section did not have higher rates of IUD expulsion and that intra‐cesarean IUD insertion was safe and effective regardless of whether the cesarean section was planned or emergent.^32^

Additionally, as mentioned above, contraceptive counseling and uptake was found to be inconsistent. In one study, they found that those seeking care for non‐emergent conditions such as for menstrual regulation left with a contraceptive method 75% of the time compared to post‐abortion patients, including those requiring MVA for removal of retained products, sepsis, or unsafe abortions, of whom 2% left the facility with contraceptive methods.^33^ In another study, among women meeting criteria for severe preexisting morbidity, only 10% received a contraceptive prescription and 50% did not even receive discussions about contraceptive services.^22^ Further, among skilled birth attendants surveyed in one study, 74% identified the need for contraceptive counseling for women treated for incomplete abortion, but only 55% emphasized the importance of referring for contraceptive methods and 44% on the need for counseling women on when they could conceive again.^40^

### 3.7 Synthesis of results

Overall, there was a dearth of literature on the subject, with only 14 studies reporting true integration of contraception and emergency obstetric care, with the vast majority referencing insertion of IUD at time of emergency cesarean section. These findings highlight the critical need for further discussion and evaluation regarding effective strategies for implementing integrated care models. Identifying best practices for integration, addressing provider hesitancies, and ensuring equitable access to contraceptive counseling and services across all EmONC settings remain pressing priorities.

## 4 DISCUSSION

Maintaining a healthy interpregnancy interval after experiencing an obstetric emergency is critical to improving maternal and neonatal outcomes. For this reason, integrating contraception into the package of EmONC services is essential to ensure timely and comprehensive care. This integration also recognizes that contraception provision is not separate from maternal health but an essential component of comprehensive care.

Overall, the findings of this scoping review highlight limited literature looking specifically at the integration of contraception at the time of EmONC services. Despite many opportunities for such discussion, including contraceptive management after postpartum hemorrhage, those with hypertensive disorders of pregnancy, emergency cesarean sections, and septic abortions, only 12 of the included studies reported directly assessing integration of IUCD or tubal ligation at the time of the emergency cesarean, with many of these studies including only a small sample size of those undergoing emergency cesarean and many not reporting subgroup‐specific outcomes. The review has also highlighted the missed opportunity in counseling high‐risk patients, with one study indicating that only 10% of women with severe preexisting morbidity received a contraceptive prescription and 50% received counseling postpartum.^32^

Evidence from the review does highlight several opportunities to enhance this integration. It is clear from this review that the literature suggests intra‐cesarean IUD insertion is safe and effective even during emergency cesarean section. It is nevertheless important to highlight that it is safe as long as contraindications are excluded particularly with regards to prolonged rupture of membranes and evidence of chorioamnionitis.^49^ Studies did not find higher rates of regret, dissatisfaction, or side effects when performed during emergency cesarean. Integrating contraceptive counseling during antenatal visits is essential to address provider concerns regarding informed consent at the time of emergency and to ensure women have had time to consider all types of contraceptive methods available. Further, ensuring postpartum contraceptives are available, and targeting provider hesitation and bias through targeted training, would be a necessary avenue for improvement.

### 4.1 Strengths

This review had several strengths, including the large research team with the ability to have frequent discussions to address bias and resolve uncertainty. All articles were reviewed by two or, if necessary, three independent reviewers to ensure inter‐rater reliability and reduce bias. Moreover, sources pulled from a wide range of databases maximized the comprehensiveness of our search. This is, to the authors' knowledge, the first scoping review in the literature considering this specific question.

### 4.2 Limitations

This study has some limitations. In addition to the general methodological challenges of a scoping review, excluding gray literature might result in an absence of insights from programmatic evaluations or governmental resources. We also excluded papers that were not written in English, which might have resulted in a more anglophone‐related understanding of the problem. Additionally, we excluded case reports in this scoping review, which might also be an alternative source for interventions. The findings are, therefore, limited to the databases we searched; however, we did include narrative and opinion pieces so long as they described the integration of EmONC and contraception at the same time or in the same intervention.

A further limitation is the fact that only papers including emergency obstetric care were included. Those papers describing routine or elective obstetric care were not analyzed. Contraceptive care is likely to be more accessible at birth in non‐emergency situations than in emergency situations. This was nevertheless out of the scope of this review but would be a subject to consider for future review and research as many lessons regarding non‐emergency provision could be adapted to use in emergency situations.

## 5 CONCLUSION

There is a dearth of literature on integrating contraception and EmONC. However, such integration has the opportunity to reduce maternal and neonatal morbidity and mortality. The findings of this paper highlight opportunities to develop relevant indicators, implementation strategies, and quality improvement initiatives to promote this integration. Efforts should focus on embedding contraception into emergency obstetric care pathways to ensure comprehensive reproductive health services and to optimize health outcomes.

## AUTHOR CONTRIBUTIONS

Reviewers: MK, AK, NE, AA, AM, HK, SM, KG, AS; Analysis: MK, AS; Manuscript first draft: MK; Manuscript editing and review: AK, NE, AA, AM, HK, SM, KG, AS.

## FUNDING INFORMATION

None.

## CONFLICT OF INTEREST STATEMENT

None.

## Supporting information


Appendix S1.

Data S1.

## Data Availability

Research data are not shared.
