# Supplementary material for: Integration of contraception provision in emergency obstetric and neonatal care: A scoping review
Source: Int J Gynaecol Obstet. 2026 Jan 5;173(3):1295–307. doi: 10.1002/ijgo.70768 (PMC13173600; doi:10.1002/ijgo.70768)
Supplement: Supplementary file 1 — Appendix S1. [file IJGO-173-1295-s002.docx]

**Appendix A- Search Strategy**

**PubMed**

| concepts | Keywords | Controlled Vocabulary (MESH) | NOTES/Full Search |
| --- | --- | --- | --- |
| Family planning | Contracepti* OR  “Postpartum contraception” OR  “intrauterine device” OR “hormonal intrauterine device” OR “copper intrauterine device” OR “tubal ligation” OR “sterilization” OR “vasectomy” OR  “Birth control shot” OR “contraceptive implant” OR “birth control implant” OR“birth control ring” OR “birth control pill” OR “oral contraceptive pill”OR “oral contraception”OR “birth control patch” OR“contraceptive patch” OR“birth control ring” OR“contraceptive ring” OR“male condom” OR“post-abortion contraception” OR “birth control”  [Title/Abstract] | "Contraception"[Mesh] OR "Intrauterine Devices"[Mesh] OR "Contraceptive Devices, Female"[Mesh] OR "Intrauterine Devices, Copper"[Mesh] OR "Intrauterine Devices, Medicated"[Mesh] OR "Sterilization, Tubal"[Mesh] OR "Sterilization, Reproductive"[Mesh] OR "Vasectomy"[Mesh] OR "Contraceptives, Oral"[Mesh] OR "Condoms"[Mesh] | "Contracepti*"[Title/Abstract] OR  "Postpartum contraception"[Title/Abstract] OR  "intrauterine device"[Title/Abstract] OR  "hormonal intrauterine device"[Title/Abstract] OR  "copper intrauterine device"[Title/Abstract] OR  "tubal ligation"[Title/Abstract] OR  "sterilization"[Title/Abstract] OR  "vasectomy"[Title/Abstract] OR  "Birth control shot"[Title/Abstract] OR  "contraceptive implant"[Title/Abstract] OR  "birth control implant"[Title/Abstract] OR  OR  "birth control pill"[Title/Abstract] OR  "oral contraceptive pill"[Title/Abstract] OR  "oral contraception"[Title/Abstract] OR  "birth control patch"[Title/Abstract] OR  "contraceptive patch"[Title/Abstract]  OR  "contraceptive ring"[Title/Abstract] OR  "male condom"[Title/Abstract] OR  "post-abortion contraception"[Title/Abstract] OR  "birth control"[Title/Abstract] OR  "Contraception"[Mesh] OR  "Intrauterine Devices"[Mesh] OR  "Contraceptive Devices, Female"[Mesh] OR  "Intrauterine Devices, Copper"[Mesh] OR  "Intrauterine Devices, Medicated"[Mesh] OR  "Sterilization, Tubal"[Mesh] OR  "Sterilization, Reproductive"[Mesh] OR  "Vasectomy"[Mesh] OR  "Contraceptives, Oral"[Mesh] OR  "Condoms"[Mesh] |
| Emergency obstetrics | "emergency obstetric and neonatal care" OR “EmONC” OR “BEmONC” OR "emergency obstetric*" OR “obstetric emergenc*” OR “obstetric hemorrhage” OR “retained placenta” OR “retained products of conception”OR “postpartum hemorrhage” OR “cesarean” OR “cesarean section” OR “eclampsia”    [Title/Abstract] | "Placenta, Retained"[Mesh] OR "Postpartum Hemorrhage"[Mesh] OR "Cesarean Section"[Mesh] OR "Eclampsia"[Mesh] | "emergency obstetric and neonatal care"[Title/Abstract] OR  "EmONC"[Title/Abstract] OR  "BEmONC"[Title/Abstract] OR  "emergency obstetric*"[Title/Abstract] OR  "obstetric emergenc*"[Title/Abstract] OR  "obstetric hemorrhage"[Title/Abstract] OR  "retained placenta"[Title/Abstract] OR  "retained products of conception"[Title/Abstract] OR  "postpartum hemorrhage"[Title/Abstract] OR  "cesarean"[Title/Abstract] OR  "cesarean section"[Title/Abstract] OR  "eclampsia"[Title/Abstract] OR  "Placenta, Retained"[Mesh] OR  "Postpartum Hemorrhage"[Mesh] OR  "Cesarean Section"[Mesh] OR  "Eclampsia"[Mesh] |

**EMBASE**

| Concept | keywords | Controlled vocab (EMTREE) | NOTES/Full Search |
| --- | --- | --- | --- |
|  | Contracepti* OR  “Postpartum contraception” OR  “intrauterine device” OR“hormonal intrauterine device”OR “copper intrauterine device” OR“tubal ligation” OR“sterilization” OR“vasectomy”OR  “Birth control shot” OR “contraceptive implant” OR“birth control implant” OR“birth control ring”OR “birth control pill” OR“oral contraceptive pill”OR “oral contraception”OR “birth control patch” OR“contraceptive patch” OR“birth control ring” OR“contraceptive ring” OR“male condom” OR“post-abortion contraception”OR “birth control”  :ti,ab | 'contraception'/exp OR 'intrauterine contraceptive device'/exp OR 'copper intrauterine device'/exp OR 'uterine tube ligation'/exp OR 'female sterilization'/exp OR 'vasectomy'/exp OR 'birth control implant'/exp OR  'oral contraceptive agent'/exp OR 'oral contraception'/exp OR 'contraceptive patch'/exp OR 'condom'/exp OR 'birth control'/exp | Contracepti*:ti,ab OR  "Postpartum contraception":ti,ab OR  "intrauterine device":ti,ab OR  "hormonal intrauterine device":ti,ab OR  "copper intrauterine device":ti,ab OR  "tubal ligation":ti,ab OR  "sterilization":ti,ab OR  "vasectomy":ti,ab OR  "Birth control shot":ti,ab OR  "contraceptive implant":ti,ab OR  "birth control implant":ti,ab OR  "birth control ring":ti,ab OR  "birth control pill":ti,ab OR  "oral contraceptive pill":ti,ab OR  "oral contraception":ti,ab OR  "birth control patch":ti,ab OR  "contraceptive patch":ti,ab OR  OR  "contraceptive ring":ti,ab OR  "male condom":ti,ab OR  "post-abortion contraception":ti,ab OR  "birth control":ti,ab OR  'contraception'/exp OR  'intrauterine contraceptive device'/exp OR  'copper intrauterine device'/exp OR  'uterine tube ligation'/exp OR  'female sterilization'/exp OR  'vasectomy'/exp OR  'birth control implant'/exp OR  'oral contraceptive agent'/exp OR  'oral contraception'/exp OR  'contraceptive patch'/exp OR  'condom'/exp OR  'birth control'/exp |
|  | "emergency obstetric and neonatal care" OR “EmONC” OR “BEmONC” OR "emergency obstetric*" OR “obstetric emergenc*” OR “obstetric hemorrhage” OR “retained placenta” OR “retained products of conception”OR “postpartum hemorrhage” OR “cesarean” OR “cesarean section” OR “eclampsia”    :ti,ab | 'obstetric hemorrhage'/exp OR 'cesarean section'/exp OR 'eclampsia'/exp OR 'obstetric emergency'/exp OR 'postpartum hemorrhage'/exp  OR 'retained placenta'/exp | "emergency obstetric and neonatal care":ti,ab OR  "EmONC":ti,ab OR  "BEmONC":ti,ab OR  "emergency obstetric*":ti,ab OR  "obstetric emergenc*":ti,ab OR  "obstetric hemorrhage":ti,ab OR  "retained placenta":ti,ab OR  "retained products of conception":ti,ab OR  "postpartum hemorrhage":ti,ab OR  "cesarean":ti,ab OR  "cesarean section":ti,ab OR  "eclampsia":ti,ab OR  'obstetric hemorrhage'/exp OR  'cesarean section'/exp OR  'eclampsia'/exp OR  'obstetric emergency'/exp OR  'postpartum hemorrhage'/exp OR  'retained placenta'/exp |

**Cinahl**

| Concept | keywords | Controlled vocab (subject headings) | NOTES/Full Search |
| --- | --- | --- | --- |
|  | Contracepti* OR  “Postpartum contraception” OR  “intrauterine device” OR“hormonal intrauterine device”OR “copper intrauterine device” OR“tubal ligation” OR“sterilization” OR“vasectomy”OR  “Birth control shot” OR“contraceptive implant” OR“birth control implant” OR“birth control ring”OR “birth control pill” OR “oral contraceptive pill”OR “oral contraception”OR “birth control patch” OR“contraceptive patch” OR“birth control ring” OR“contraceptive ring” OR“male condom” OR“post-abortion contraception”OR “birth control”    TI ("family planning") OR AB ("family planning") | (MH "Contraception+") OR  (MH "Intrauterine Devices") OR (MH "Sterilization, Tubal") OR (MH "Vasectomy") OR (MH "Contraceptives, Oral Combined") OR (MH "Contraceptives, Oral+")  OR (MH "Condoms+") | TI ("Contracepti*") OR AB ("Contracepti*") OR  TI ("Postpartum contraception") OR AB ("Postpartum contraception") OR  TI ("intrauterine device") OR AB ("intrauterine device") OR  TI ("hormonal intrauterine device") OR AB ("hormonal intrauterine device") OR  TI ("copper intrauterine device") OR AB ("copper intrauterine device") OR  TI ("tubal ligation") OR AB ("tubal ligation") OR  TI ("sterilization") OR AB ("sterilization") OR  TI ("vasectomy") OR AB ("vasectomy") OR  TI ("Birth control shot") OR AB ("Birth control shot") OR  TI ("contraceptive implant") OR AB ("contraceptive implant") OR  TI ("birth control implant") OR AB ("birth control implant") OR  TI ("birth control ring") OR AB ("birth control ring") OR  TI ("birth control pill") OR AB ("birth control pill") OR  TI ("oral contraceptive pill") OR AB ("oral contraceptive pill") OR  TI ("oral contraception") OR AB ("oral contraception") OR  TI ("birth control patch") OR AB ("birth control patch") OR  TI ("contraceptive patch") OR AB ("contraceptive patch") OR  TI ("contraceptive ring") OR AB ("contraceptive ring") OR  TI ("male condom") OR AB ("male condom") OR  TI ("post-abortion contraception") OR AB ("post-abortion contraception") OR  TI ("birth control") OR AB ("birth control") OR  (MH "Contraception+") OR  (MH "Intrauterine Devices") OR  (MH "Sterilization, Tubal") OR  (MH "Vasectomy") OR  (MH "Contraceptives, Oral Combined") OR  (MH "Contraceptives, Oral+") OR  (MH "Condoms+") |
|  | "emergency obstetric and neonatal care" OR “EmONC” OR “BEmONC” OR "emergency obstetric*" OR “obstetric emergenc*” OR “obstetric hemorrhage” OR “retained placenta” OR “retained products of conception”OR “postpartum hemorrhage” OR “cesarean” OR “cesarean section” OR “eclampsia”  TI ("family planning") OR AB ("family planning") | (MH "Cesarean Section+") OR (MH "Eclampsia+") OR (MH "Obstetric Emergencies") OR (MH "Postpartum Hemorrhage") OR (MH "Placenta, Retained") | TI ("emergency obstetric and neonatal care") OR AB ("emergency obstetric and neonatal care") OR  TI ("EmONC") OR AB ("EmONC") OR  TI ("BEmONC") OR AB ("BEmONC") OR  TI ("emergency obstetric*") OR AB ("emergency obstetric*") OR  TI ("obstetric emergenc*") OR AB ("obstetric emergenc*") OR  TI ("obstetric hemorrhage") OR AB ("obstetric hemorrhage") OR  TI ("retained placenta") OR AB ("retained placenta") OR  TI ("retained products of conception") OR AB ("retained products of conception") OR  TI ("postpartum hemorrhage") OR AB ("postpartum hemorrhage") OR  TI ("cesarean") OR AB ("cesarean") OR  TI ("cesarean section") OR AB ("cesarean section") OR  TI ("eclampsia") OR AB ("eclampsia") OR  (MH "Cesarean Section+") OR  (MH "Eclampsia+") OR  (MH "Obstetric Emergencies") OR  (MH "Postpartum Hemorrhage") OR  (MH "Placenta, Retained") |

**Web of Science**

| Concept | keywords | NOTES/Full Search |
| --- | --- | --- |
|  | Contracepti* OR  “Postpartum contraception” OR  “intrauterine device” OR“hormonal intrauterine device”OR “copper intrauterine device” OR“tubal ligation” OR“sterilization” OR“vasectomy”OR  “Birth control shot” OR“contraceptive implant” OR“birth control implant” OR“birth control ring”OR “birth control pill” OR“oral contraceptive pill”OR “oral contraception”OR “birth control patch” OR“contraceptive patch” OR“birth control ring” OR“contraceptive ring” OR“male condom” OR“post-abortion contraception”OR “birth control” | Contracepti* OR  “Postpartum contraception”  OR  “intrauterine device”  OR  “hormonal intrauterine device”  OR  “copper intrauterine device”  OR  “tubal ligation”  OR  “sterilization”  OR  “vasectomy”  OR  “Birth control shot”  OR  “contraceptive implant”  OR  “birth control implant”  OR  “birth control ring”  OR  “birth control pill”  OR  “oral contraceptive pill”  OR  “oral contraception”  OR  “birth control patch”  OR  “contraceptive patch”  OR  “contraceptive ring”  OR  “male condom”  OR  “post-abortion contraception”  OR  “birth control” |
|  | "emergency obstetric and neonatal care" OR “EmONC” OR “BEmONC” OR "emergency obstetric*" OR “obstetric emergenc*” OR “obstetric hemorrhage” OR “retained placenta” OR “retained products of conception”OR “postpartum hemorrhage” OR “cesarean” OR “cesarean section” OR “eclampsia” | "emergency obstetric and neonatal care"  OR  “EmONC”  OR  “BEmONC”  OR  "emergency obstetric*"  OR  “obstetric emergenc*”  OR  “obstetric hemorrhage”  OR  “retained placenta”  OR  “retained products of conception”  OR  “postpartum hemorrhage”  OR  “cesarean”  OR  “cesarean section”  OR  “eclampsia” |

**Cochrane Library**

| Concept | keywords | Controlled vocab | NOTES/Full Search |
| --- | --- | --- | --- |
|  | Contracepti* OR  “Postpartum contraception” OR  “intrauterine device” OR“hormonal intrauterine device”OR “copper intrauterine device” OR“tubal ligation” OR“sterilization” OR“vasectomy”OR  “Birth control shot” OR“contraceptive implant” OR“birth control implant” OR“birth control ring”OR “birth control pill” OR“oral contraceptive pill”OR “oral contraception”OR “birth control patch” OR“contraceptive patch” OR“birth control ring” OR“contraceptive ring” OR“male condom” OR“post-abortion contraception”OR “birth control” | "Contraception"[Mesh] OR "Intrauterine Devices"[Mesh] OR "Contraceptive Devices, Female"[Mesh] OR "Intrauterine Devices, Copper"[Mesh] OR "Intrauterine Devices, Medicated"[Mesh] OR "Sterilization, Tubal"[Mesh] OR "Sterilization, Reproductive"[Mesh] OR "Vasectomy"[Mesh] OR "Contraceptives, Oral"[Mesh] OR "Condoms"[Mesh] |  |
|  | "emergency obstetric and neonatal care" OR “EmONC” OR “BEmONC” OR "emergency obstetric*" OR “obstetric emergenc*” OR “obstetric hemorrhage” OR “retained placenta” OR “retained products of conception”OR “postpartum hemorrhage” OR “cesarean” OR “cesarean section” OR “eclampsia” | "Placenta, Retained"[Mesh] OR "Postpartum Hemorrhage"[Mesh] OR "Cesarean Section"[Mesh] OR "Eclampsia"[Mesh] |  |

**Appendix B- Data tool**

**General:**

- Study ID
- Title
- Author
- Year Published
- Country/Countries
- Setting (hospital, clinic, other)

**Methods**

- Aim of study (1 sentence description of study objectives/ if not a study, include purpose of the review or commentary)
- Study design (RCT, non randomized experimental study, cohort, cross sectional, case control, systematic review, qualitative research, text and opinion, narrative review, commentary, other)
- Population description
- Total number of participants

**Results**

- Type of contraceptive services offered (contraceptive provision, counseling, other)
- Types of contraceptives offered (IUD copper, IUD hormonal, pill, implant, other)
- Nature of EmONC services (emergency csection, hemorrhage, sepsis, hypertensive disorder, other)
  - If not a study, describe the scenario in which Emonc integration was discussed
- Did integration actually happen (Yes, No a hypothetical scenario was discussed, N general recommendations were provided, Implementation Plan, Other)
- Number of participants who received contraceptives at time of emergency

**Barriers to integration**: (Please note if there were any specific challenges to integrating contraceptives AT TIME of emergency mentioned in the article)

**Facilitators of integration:** (Please note if there were any specific facilitators to integrating contraceptives AT TIME of emergency mentioned in the article)

**Additional Comments** (anything important that you would like to add that is not captured in the above data)
